# Nitrogen Removal Characteristics of* Pseudomonas putida* Y-9 Capable of Heterotrophic Nitrification and Aerobic Denitrification at Low Temperature

**DOI:** 10.1155/2017/1429018

**Published:** 2017-02-15

**Authors:** Yi Xu, Tengxia He, Zhenlun Li, Qing Ye, Yanli Chen, Enyu Xie, Xue Zhang

**Affiliations:** Chongqing Key Laboratory of Soil Multiscale Interfacial Process, Southwest University, Chongqing 400716, China

## Abstract

The cold-adapted bacterium* Pseudomonas putida* Y-9 was investigated and exhibited excellent capability for nitrogen removal at 15°C. The strain capable of heterotrophic nitrification and aerobic denitrification could efficiently remove ammonium, nitrate, and nitrite at an average removal rate of 2.85 mg, 1.60 mg, and 1.83 mg NL^−1 ^h^−1^, respectively. Strain Y-9 performed nitrification in preference to denitrification when ammonium and nitrate or ammonium and nitrite coexisted in the solution. Meantime, the presence of nitrate had no effect on the ammonium removal rate of strain Y-9, and yet the presence of high concentration of nitrite would inhibit the cell growth and decrease the nitrification rate. The experimental results indicate that* P. putida* Y-9 has potential application for the treatment of wastewater containing high concentrations of ammonium along with its oxidation products at low temperature.

## 1. Introduction

Excess nitrogen concentration, often in the form of nitrate and ammonium, would lead to eutrophication of water. And this would affect the balance of natural water ecosystems, even human health [1]. Therefore, excessive nitrogen has become a growing concern. In recent years, biological treatment is recognized as the prevailing method because of its high efficiency, lower maintenance costs, and environmental friendliness. Conventional processes of ammonium removal involved nitrification by aerobic autotrophic organisms and denitrification by anaerobic heterotrophic organisms. However, such systems are time-consuming and inconvenience for management and operation due to the low rate of nitrification and the complexity of separating aerobic and anoxic tanks [2]. To date, microbes which have the ability to convert ammonium compounds to gaseous products and remove organic matter simultaneously under aerobic conditions are found and isolated. That is to say, these microorganisms, such as* Providencia rettgeri* YL [3],* Bacillus methylotrophicus* strain L7 [4],* Alcaligenes faecalis* strain number 4 [5], and* V. diabolicus* SF16 [6], have the ability to perform simultaneously heterotrophic nitrification and aerobic denitrification in a single reactor, which will reduce the space demand and improve the efficiency of nitrogen removal significantly [7].

Nevertheless, the growth and bioactivity of microorganisms are affected significantly by temperature. Previous research has shown that both nitrification and denitrification would be inhibited strongly at temperatures below 20°C [8, 9]. Most of the denitrifiers reported previously can only have potential applications for wastewater treatment systems at moderate temperature. Thus, these denitrifiers cannot be applied to wastewater treatment at cold conditions because of their too sensitivity to low temperature. In addition, accumulation of nitrite or nitrate, which has potential threat to the safety of water zoology and drinking water due to their high toxicity, was observed during heterotrophic nitrification [10–12]. In that case, it is urgent to discover more denitrifiers capable of adapting to low temperature and producing less intermediate.

In our previous study [13], a novel hypothermia aerobic nitrite-denitrifying bacterium, strain Y-9, was isolated from the long-term flooded paddy soil and identified as* Pseudomonas putida*. And* P. Putida* Y-9 exhibited an excellent performance of denitrification with a low concentration of nitrite at low temperature. But its ability of heterotrophic nitrification and aerobic denitrification with other nitrogen compounds was unclear. In the study, the nitrogen removal performance of strain Y-9 with high concentrations of ammonium, nitrate, nitrite, and organic nitrogen was investigated at 15°C, and the characteristics of nitrification and denitrification were assessed by analyzing the intermediate products during the nitrification and denitrification process. The results indicated that strain Y-9 is the heterotrophic nitrifying-aerobic denitrifying psychrophile bacteria and could quickly remove high concentration ammonium in wastewater. Although several strains belong to* P. putida* are reported as heterotrophic nitrifier [14] or aerobic denitrifier [15], they are all mesophilic bacteria. To the best of our knowledge, this is the first report about* P. putida* capable of heterotrophic nitrification and aerobic denitrification at low temperature. All attempts above would be applied to elucidate the nitrogen removal characteristics of strain Y-9. And strain Y-9 might enhance aerobic denitrification activity of indigenous bacteria by quickly decreasing ammonium concentration in wastewater, especially cold wastewater.

## 2. Materials and Methods

### 2.1. Microorganism

Strain Y-9 was isolated from the long-term flooded paddy soil and successfully identified as* Pseudomonas putida* and used for the investigation on nitrogen removal performance.

### 2.2. Media

Heterotrophic nitrification medium (HNM) [16] contained (per liter) 7.0 g K_2_HPO_4_, 3.0 g KH_2_PO_4_, 0.1 g MgSO_4_·7H_2_O, 1.0 g (NH_4_)_2_SO_4_, 0.05 g FeSO_4_·7H_2_O, and 10 g CH_3_COONa, pH 7.2. HNM was used to measure the nitrification ability of strain Y-9. Aerobic denitrification medium (ADM) contained (per liter) 7.0 g K_2_HPO_4_, 3.0 g KH_2_PO_4_, 0.1 g MgSO_4_·7H_2_O, 1.45 g KNO_3_ (ADM-1) or 0.986 g NaNO_2_ (ADM-2), 0.05 g FeSO_4_·7H_2_O, and 10 g CH_3_COONa, pH 7.2. ADM was used to test the aerobic denitrification ability of strain Y-9. Tryptone (1.575 g/L) was used instead of inorganic nitrogen in the ADM to detect the aerobic denitrification ability of strain Y-9 with organic nitrogen. Simultaneous nitrification and denitrification medium (SNDM) consisted of the following components (per liter): 7.0 g K_2_HPO_4_, 3.0 g KH_2_PO_4_, 0.1 g MgSO_4_·7H_2_O, 1.0 g (NH_4_)_2_SO_4_, 1.45 g KNO_3_ (SNDM-1) or 0.986 g NaNO_2_ (SNDM-2), 0.05 g FeSO_4_·7H_2_O, and 10 g CH_3_COONa, pH 7.2. SNDM-1 and SNDM-2 were used to analyze simultaneous heterotrophic nitrification and aerobic denitrification capability of strain Y-9. Luria-Bertani (LB) medium contained (per liter) 10 g tryptone, 5 g yeast extract, and 10 g NaCl, pH 7.5.

All chemicals were of analytical grade. And conical flasks (250 ml capacity) containing 100 ml medium was autoclaved for 30 min at 121°C.

### 2.3. Assessment of Nitrogen Removal Capability with Different Nitrogen Source

Single colony of Y-9 was cultivated for 36 h in 100 ml sterile LB broth medium at 15°C and 150 rpm. After 36 h cultivation, the strain cell in 8 ml cultivation medium was harvested by centrifuging at 4000 rpm for 8 min and washed once with sterile water. The pellets were inoculated into 100 ml HNM, ADM-1, ADM-2, organic medium, SNDM-1, or SNDM-2, respectively. The cultures were incubated at 15°C with 150 rpm shaking speed for 4 days. And the medium without inoculation was used as control. During incubation, the different cultures were sampled to determine the concentration of NH_4_^+^-N, NO_2_^−^-N, NO_3_-N, TN, and optical density (OD_600_) at 24 intervals. All experiments were conducted in triplicate.

### 2.4. Analytical Methods

Cell density was tested OD_600_ by using a spectrophotometer (DU800, Beckman Coulter, USA). Total nitrogen was calculated by the absorbance value at 220 nm subtracting the two times background absorbance value at 275 nm after using alkaline potassium persulfate digestion. Ammonium, nitrate, and nitrite were detected using the supernatant after samples centrifuged at 8000 rpm for 5 min. Ammonium nitrogen was analyzed by indophenols blue method. Nitrate was calculated by the absorbance value at 220 nm subtracting the two times background absorbance value at 275 nm. The concentration of NO_2_^−^-N was determined by N-(1-naphthalene)-diaminoethane spectrophotometry method according to the State Environmental Protection Administration of China [14].

### 2.5. Statistical Analysis and Graphical Work

Statistical analysis and graphical work were carried out by using Excel, SPSS Statistics, and Origin 8.6. The results were presented as means ± SD (standard deviation of means).

## 3. Results and Discussions

### 3.1. Ammonium Removal Performance of Strain Y-9 at Low Temperature

**Figure 1:**
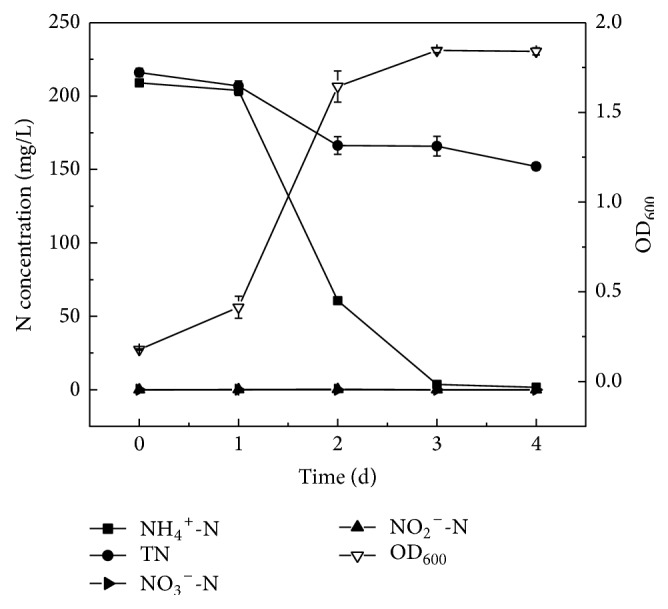
The growth curve and ammonium removal performance of strain Y-9 in the HNM at 15°C.

To assess heterotrophic nitrification with ammonium of strain Y-9 at low temperature, strain Y-9 was cultivated in the HNM at 15°C. The growth curve and ammonium removal capability of strain Y-9 were shown in Figure 1. Strain Y-9 grew quickly from 1 d to 2 d, and OD_600_ reached 1.644 at 2 d, which was higher than that of* Pseudomonas fluorescens* wsw-1001 at 48 h with an OD_600_ of 0.554 under the same conditions [15]. This showed that strain Y-9 well adapted to low temperatures. Subsequently, strain Y-9 reached the stationary phase. After 3 d of incubation, the concentration of ammonium nitrogen decreased dramatically from 208.94 mg/L to 3.56 mg/L, and 98.3% of ammonium removal was achieved. The average nitrification rate of strain Y-9 was approximately 2.85 mg NH_4_^+^-N/L/h, which was observably higher than that of heterotrophic nitrification-aerobic denitrification bacterium* Aeromonas* sp. HN-02 possessed an average rate of 2.32 mg NH_4_^+^-N/L/h at the same temperature [17].

Furthermore, it was attractive that ammonium removal performance of strain Y-9 at low temperature was even better than most bacterial at moderate temperature (around 30°C), such as* P. alcaligenes* AS-1 (1.15 mg NH_4_^+^-N/L/h) [18],* Diaphorobacter* sp. PD-7 (1.61 mg NH_4_^+^-N/L/h) [19], and* Marinobacter* strain NNA5 (1.23 mg NH_4_^+^-N/L/h) [20]. The concentration of total nitrogen reduced observably from 216.10 mg/L to 152.08 mg/L, and the removal efficiency was 29.6% during ammonium removal process. Meanwhile, a maximum NO_2_^−^-N concentration of 0.31 mg/L was detected and then declined, and nitrate was undetectable during the whole process. This would be a significant advantage in practical application of ammonium removal to avoid high accumulation of nitrification products under low temperature. Additionally, the production of gaseous nitrogen by strain Y-9 might be via the intermediate nitrite when ammonium was used as the sole nitrogen source. Similar result had been reported on* Vibrio diabolicus* SF16 [6] and* Zobellella taiwanensis* DN-7 [21] in which the intermediate nitrite was only detected in trace amounts and nitrate was not observed during heterotrophic nitrification. Nevertheless, nitrite was undetected, and trace accumulation of nitrate was observed during the ammonium removal process by* Acinetobacter* sp. Y16 [22]. In addition, dominant accumulation of nitrite and nitrate were produced by* Rhodococcus* sp. CPZ24 [23],* Marinobacter* sp. F6 [24], and* Chryseobacterium* sp. R31 [2] in the process of nitrification. The experimental results indicated that strain Y-9 could efficiently utilize ammonium to conduct nitrification coupled with aerobic denitrification at low temperature, which is of great benefit to enhance aerobic denitrification activity of indigenous bacteria by quickly decreasing ammonium concentration in wastewater, especially cold wastewater.

### 3.2. Nitrate Removal Performance of Strain Y-9 at Low Temperature

**Figure 2:**
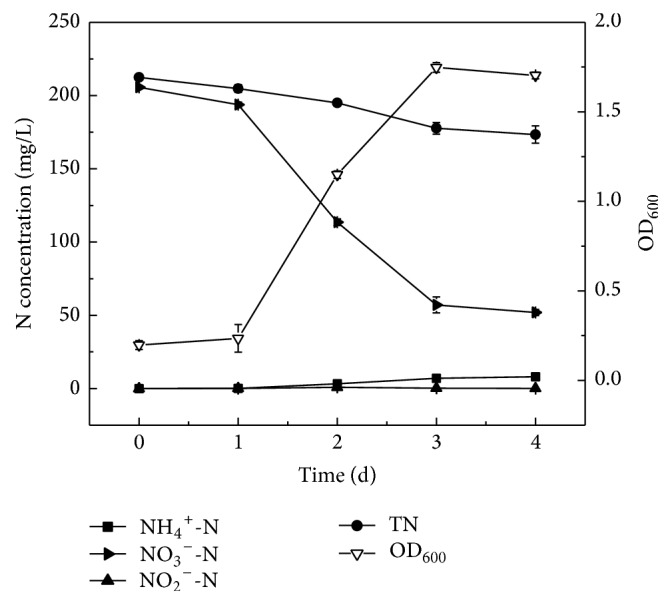
The growth curve and nitrate removal performance of strain Y-9 in the ADM-1 at 15°C.

To demonstrate the denitrification by strain Y-9 at low temperature, KNO_3_ was used as the sole nitrogen source in the ADM-1 at 15°C.  Figure 2 showed the growth curve and nitrate removal characteristic of strain Y-9. A significant decrease of nitrate was observed from 1 d to 3 d as the OD_600_ increased from 0.235 to 1.748, and cell growth reached the stationary phase after 3 d. By 4 d of cultivation, approximately 74.7% of nitrate nitrogen (205.63 mg/L initial NO_3_^−^-N) and 18.3% of total nitrogen (212.36 mg/L initial TN) were removed. The average denitrification rate of strain Y-9 was 1.60 mg NO_3_^−^-N/L/h, which was similar to that of* P. migulae* AN-1 which possessed an average rate of 1.57 mg NO_3_^−^-N/L/h at 10°C [25]. Meanwhile, nitrite was only detected in trace amounts, which was discrepant from the report that the dominant accumulation of nitrite was observed during the removal of nitrate [7, 26]. This may be due to the high nitrite reductase activity of strain Y-9.

In addition, ammonium nitrogen gradually increased from 1 d to 4 d, and the final concentration was approximately 8.04 mg/L. This phenomenon was different from the report that the production of ammonium might be the decomposition of cell, and part of nitrogen containing in the cell was released into the medium in senescent phases [27]. Therefore, the process of dissimilatory nitrate reduction to ammonium (DNRA) might present in strain Y-9, but this requires further investigation. In previous reports, Su et al. [28] firstly reported that* P. alcaliphila* strain MBR could conduct denitrification and DNRA simultaneously with an electrode as the sole electron donor in bioelectrochemical systems (BESs), but not in an open culture. The experimental results indicated that strain Y-9 could perform denitrification with nitrate at low temperature.

### 3.3. Nitrite Removal Performance of Strain Y-9 at Low Temperature

**Figure 3:**
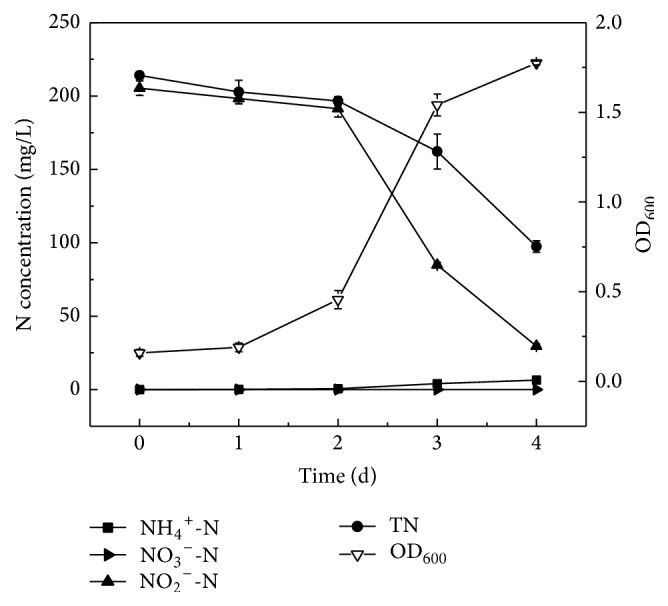
The growth curve and nitrite removal performance of strain Y-9 in the ADM-2 at 15°C.

Denitrification performance of strain Y-9 with nitrite (NaNO_2_) as the sole nitrogen source was evaluated.  Figure 3 showed the growth curve and nitrite removal characteristic of strain Y-9 in the ADM-2 at 15°C. A significant decrease in NO_2_^−^-N by strain Y-9 was observed. The growth of strain Y-9 slowly increased at the initial 2 d. Subsequently, the NO_2_^−^-N was rapidly reduced, and OD_600_ reached 1.775 at 4 d. By 4 d of cultivation, the concentration of total nitrogen decreased dramatically from 214.12 mg/L to 97.45 mg/L, and approximately 54.5% of total nitrogen was removed. Meantime, 85.6% of nitrite (205.32 mg/L initial NO_2_^−^-N) was removed. The average denitrification rate of strain Y-9 was approximately 1.83 mg NO_2_^−^-N/L/h, which was higher than that of* Pseudomonas *sp. yy7 which possessed an average rate of 0.76 mg NO_2_^−^-N/L/h [29]. Nitrate was undetectable during the whole process.

The concentration of ammonium nitrogen gradually increased to 6.43 mg/L from 1 d to 4 d. Similarly, the process of dissimilatory nitrite reduction to ammonium might occur in strain Y-9. Moreover, the higher nitrogen removal efficiency was achieved with nitrite as the nitrogen source compared with nitrate. The similar performance was reported on strain 1 [30] and* P. migulae* AN-1 [25]. On the contrary, nitrite reduction rate of* Bacillus *sp. LY [31] and* Pseudomonas stutzeri* T13 [32] was much lower than the nitrate removal rate under aerobic conditions. The results above showed that strain Y-9 exhibited the higher removal performance on nitrite compared with nitrate at low temperature.

### 3.4. Assessment of Organic Nitrogen Removal Performance by Strain Y-9 at Low Temperature

**Figure 4:**
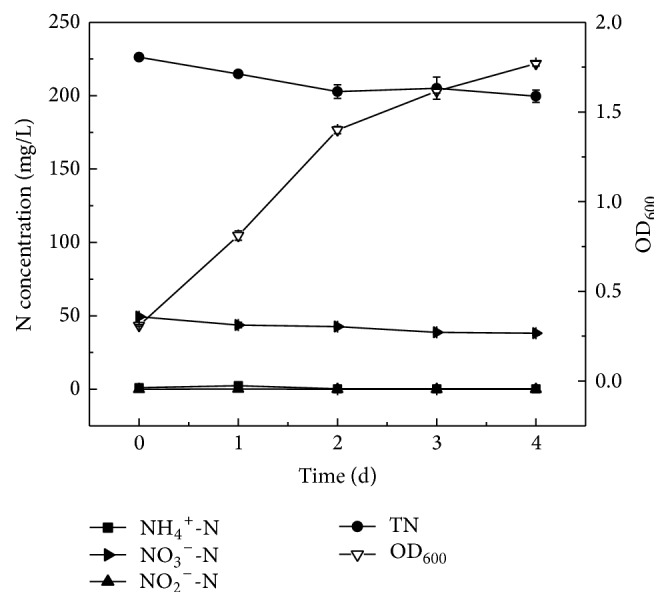
The growth curve and nitrogen removal performance of strain Y-9 with tryptone as the organic nitrogen source at 15°C.

Organic nitrogen is a kind of pollutant in the wastewater. In the present study, strain Y-9 grew well in the organic nitrogen medium at low temperature.  Figure 4 illustrated the cell growth curve and nitrogen removal characteristics of strain Y-9 with tryptone as the organic nitrogen source at 15°C. Tryptone is a compound containing amino acids, ammonium, nitrate, and so on. Strain Y-9 grew well in the medium, and OD_600_ gradually increased to 1.77 at 4 d. By 4 d of cultivation, 26.7 mg/L of total nitrogen was removed, and approximately 11.8% of total nitrogen was removed, which denoted that strain Y-9 could utilize tryptone for cell growth, but hardly convert it to nitrogenous gas.

### 3.5. Assessment of Simultaneous Nitrification and Denitrification Performance by Strain Y-9 in the SNDM-1 at Low Temperature

**Figure 5:**
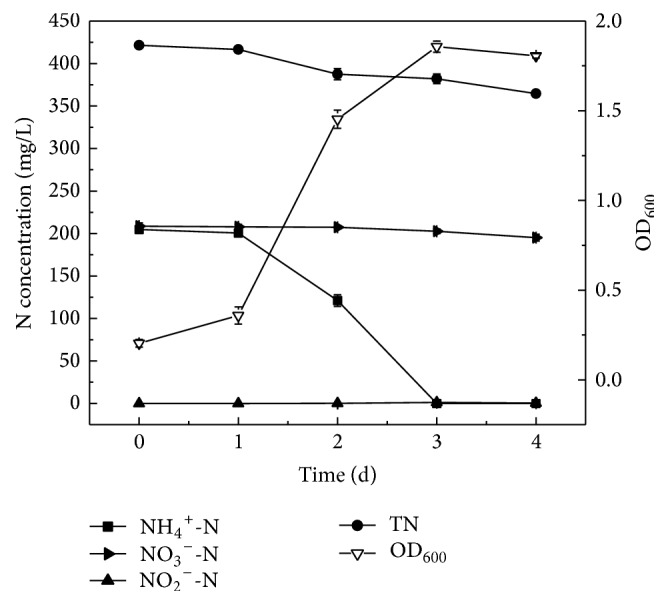
The growth curve and nitrogen removal performance of strain Y-9 in the SNDM-1 at 15°C.

Biological simultaneous nitrification and denitrification is a more efficient and economic method for nitrogen removal from wastewater [33]. Strain Y-9 could perform heterotrophic nitrification and denitrification separately under aerobic conditions. To evaluate aerobic simultaneous nitrification and denitrification performance of strain Y-9 at low temperature, strain Y-9 was cultivated in the SNDM-1 with (NH_4_)_2_SO_4_ and KNO_3_ as the nitrogen sources at 15°C.  Figure 5 showed the growth curve and nitrogen removal characteristics of strain Y-9 at 15°C. Strain Y-9 grew quickly from 1 d to 3 d and then reached the stationary phase. In the meantime, the concentration of ammonium decreased dramatically during the log phase. Furthermore, approximately 100% of ammonium nitrogen (204.72 mg/L initial NH_4_^+^-N) was completely removed after 3 d of cultivation, and the average nitrification rate of strain Y-9 was 2.84 mg NH_4_^+^-N/L/h, which was almost equivalent to that of ammonium as the sole nitrogen source. Thus, the presence of nitrate had no material impact on the nitrification rate of strain Y-9, which was obviously different from previous report that the presence of nitrate could decrease the nitrification rate [34].

Nevertheless, the removal rate of nitrate was far lower than that of nitrate as the single nitrogen source, and removal efficiency of nitrate was only 6.4% after 4 d of cultivation. This phenomenon was consistent with the report that* Rhodococcus* sp. HY-1 utilized ammonium preferentially when ammonium and nitrate coexisted in the medium, and nitrate could be utilized when ammonium was completely exhausted under aerobic conditions. However, this result was different from the report that* Klebsiella pneumoniae* CF-S9 had the ability to achieve simultaneous removal of ammonium and nitrate, and nitrate removal rate was higher than that of ammonium when ammonium and nitrate coexisted in the medium [35]. There was only trace accumulation of nitrite, and about 56.82 mg/L of TN was converted to gaseous nitrogen during the nitrification and denitrification process of strain Y-9. All above results indicated that strain Y-9 conducted nitrification in preference to denitrification when ammonium and nitrate coexisted in the medium under aerobic conditions at low temperature.

### 3.6. Assessment of Simultaneous Nitrification and Denitrification Performance by Strain Y-9 in the SNDM-2 at Low Temperature

**Figure 6:**
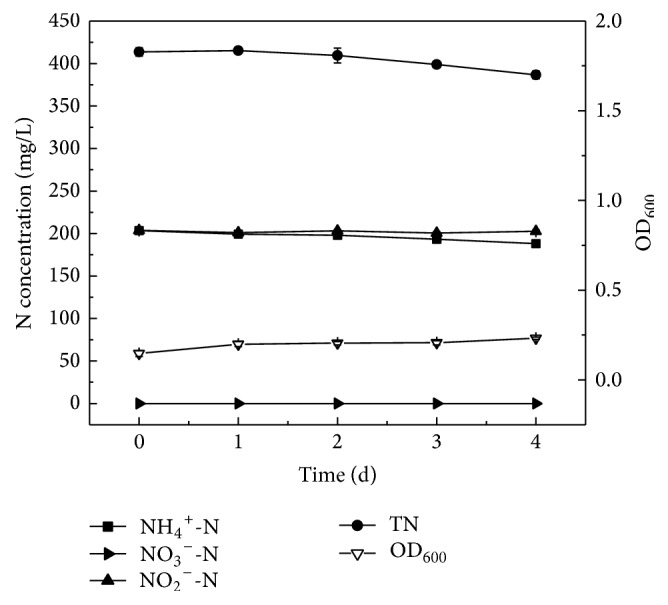
The growth curve and nitrogen removal performance of strain Y-9 in the SNDM-2 at 15°C.

The capability of simultaneous nitrification and denitrification by strain Y-9 with (NH_4_)_2_SO_4_ and NaNO_2_ as the nitrogen sources at 15°C was plotted in Figure 6. Strain Y-9 grew very slowly, and OD_600_ only slightly increased to 0.232 at 4 d, which correlated well with the low nitrogen removal efficiency. Hence, the concentration of ammonium nitrogen decreased inconspicuously from 203.43 mg/L to 188.13 mg/L, and only 7.5% of ammonium was removed after 4 d of cultivation. The average nitrification rate of strain Y-9 was 0.16 mg NH_4_^+^-N/L/h, which was much less than that of ammonium or nitrite as the nitrogen source. Moreover, nitrate was undetectable during the whole process, and 203.58 mg/L initial NO_2_^−^-N was almost unchanged, which was inconsistent with the report that the presence of ammonium together with nitrite could promote the nitrite removal efficiency by* Marichromatium gracile* YL28 [34] or promote the ammonium removal efficiency by* Pseudomonas* sp. qy37 [36]. This phenomenon was consistent with the report that the nitrification rate of* A. Faecalis* TUD would be inhibited from the presence of nitrite [37]. However, nitrogen removal performance of strain Y-9 was contrary to* P. versutus* LYM, which could synchronize ammonium removal with nitrite removal and could not reduce nitrite when nitrite was used as the sole nitrogen source even in presence of enough carbon source [38].

Strain Y-9 could simultaneously reduce ammonium and its nitrification product (nitrite) when ammonium was used as the sole nitrogen source. But ammonium and adscititious nitrite could not be removed synchronously by strain Y-9. This might be explained in that enzyme activity (such as ammonia monooxygenase) related to the nitrification could be inhibited from the presence of high concentration of nitrite, thereby affecting the ammonium nitrogen removal. The results indicated that the presence of high concentration of nitrite would restrain the cell growth and decrease the ammonium removal.

## 4. Conclusion

In this study,* Pseudomonas putida* Y-9 could perform heterotrophic nitrification with ammonium and aerobic denitrification with nitrate or nitrite at 15°C. The strain could efficiently remove ammonium, nitrate, and nitrite at average removal rate of 2.85 mg, 1.60 mg, and 1.83 mg NL^−1 ^h^−1^, respectively. Strain Y-9 performed nitrification in preference to denitrification when ammonium and nitrate or ammonium and nitrite coexisted in the solution. Meantime, the presence of nitrate had no impact on the nitrification rate of strain Y-9. However, the presence of high concentration of nitrite could suppress cell growth and lower the nitrification rate. In addition, the process of dissimilatory nitrate or nitrite reduction to ammonium (DNRA) might present in strain Y-9. The results indicated that* P. putida* Y-9 has potential application for wastewater treatment of nitrogen pollution under cold temperature conditions. What is more, the nitrogen transformation mechanism of strain Y-9 needs further research.

## References

[1] Shi Z., Zhang Y., Zhou J., Chen M., Wang X. (2013). Biological removal of nitrate and ammonium under aerobic atmosphere by *Paracoccus versutus* LYM. *Bioresource Technology*.

[2] Kundu P., Pramanik A., Dasgupta A., Mukherjee S., Mukherjee J. (2014). Simultaneous heterotrophic nitrification and aerobic denitrification by *Chryseobacterium* sp. R31 isolated from abattoir wastewater. *BioMed Research International*.

[3] Taylor S. M., He Y., Zhao B., Huang J. (2009). Heterotrophic ammonium removal characteristics of an aerobic heterotrophic nitrifying-denitrifying bacterium, *Providencia rettgeri* YL. *Journal of Environmental Sciences*.

[4] Zhang Q.-L., Liu Y., Ai G.-M., Miao L.-L., Zheng H.-Y., Liu Z.-P. (2012). The characteristics of a novel heterotrophic nitrification-aerobic denitrification bacterium, *Bacillus methylotrophicus* strain L7. *Bioresource Technology*.

[5] Shoda M., Ishikawa Y. (2014). Heterotrophic nitrification and aerobic denitrification of high-strength ammonium in anaerobically digested sludge by *Alcaligenes faecalis* strain No. 4. *Journal of Bioscience and Bioengineering*.

[6] Duan J., Fang H., Su B., Chen J., Lin J. (2015). Characterization of a halophilic heterotrophic nitrification-aerobic denitrification bacterium and its application on treatment of saline wastewater. *Bioresource Technology*.

[7] Zhou M., Ye H., Zhao X. (2014). Isolation and characterization of a novel heterotrophic nitrifying and aerobic denitrifying bacterium *Pseudomonas stutzeri* KTB for bioremediation of wastewater. *Biotechnology and Bioprocess Engineering*.

[8] Zheng H., Liu Y., Sun G., Gao X., Zhang Q., Liu Z. (2011). Denitrification characteristics of a marine origin psychrophilic aerobic denitrifying bacterium. *Journal of Environmental Sciences*.

[9] Yao S., Ni J., Ma T., Li C. (2013). Heterotrophic nitrification and aerobic denitrification at low temperature by a newly isolated bacterium, *Acinetobacter* sp. HA2. *Bioresource Technology*.

[10] Chen Q., Ni J. (2011). Heterotrophic nitrification-aerobic denitrification by novel isolated bacteria. *Journal of Industrial Microbiology and Biotechnology*.

[11] Zhao B., An Q., He Y. L., Guo J. S. (2012). N_2_O and N_2_ production during heterotrophic nitrification by *Alcaligenes faecalis* strain NR. *Bioresource Technology*.

[12] Liu Y., Wang Y., Li Y., An H., Lv Y. (2015). Nitrogen removal characteristics of heterotrophic nitrification-aerobic denitrification by *Alcaligenes faecalis* C16. *Chinese Journal of Chemical Engineering*.

[13] He T. X., Li Z. L. (2015). Identification and denitrification characterization of a psychrotrophic and aerobic nitrite-bacterium. *Biotechnology Bulletin*.

[14] State Environmental Protection Administration of China (2012). *Water and Wastewater Analysis Methods*.

[15] Zhang S., Sha C., Jiang W. (2015). Ammonium removal at low temperature by a newly isolated heterotrophic nitrifying and aerobic denitrifying bacterium *Pseudomonas fluorescens* wsw-1001. *Environmental Technology*.

[16] Pal R. R., Khardenavis A. A., Purohit H. J. (2015). Identification and monitoring of nitrification and denitrification genes in *Klebsiella pneumoniae* EGD-HP19-C for its ability to perform heterotrophic nitrification and aerobic denitrification. *Functional and Integrative Genomics*.

[17] Chen M., Wang W., Feng Y. (2014). Impact resistance of different factors on ammonia removal by heterotrophic nitrification-aerobic denitrification bacterium *Aeromonas*sp. HN-02. *Bioresource Technology*.

[18] Su J.-J., Yeh K.-S., Tseng P.-W. (2006). A strain of *Pseudomonas*sp. isolated from piggery wastewater treatment systems with heterotrophic nitrification capability in Taiwan. *Current Microbiology*.

[19] Ge Q., Yue X., Wang G. (2015). Simultaneous heterotrophic nitrification and aerobic denitrification at high initial phenol concentration by isolated bacterium *Diaphorobacter* sp. PD-7. *Chinese Journal of Chemical Engineering*.

[20] Liu Y., Ai G.-M., Miao L.-L., Liu Z.-P. (2016). *Marinobacter* strain NNA5, a newly isolated and highly efficient aerobic denitrifier with zero N_2_O emission. *Bioresource Technology*.

[21] Lei Y., Wang Y., Liu H., Xi C., Song L. (2016). A novel heterotrophic nitrifying and aerobic denitrifying bacterium, *Zobellella taiwanensis* DN-7, can remove high-strength ammonium. *Applied Microbiology and Biotechnology*.

[22] Huang X., Li W., Zhang D., Qin W. (2013). Ammonium removal by a novel *oligotrophic Acinetobacter* sp. Y16 capable of heterotrophic nitrification-aerobic denitrification at low temperature. *Bioresource Technology*.

[23] Chen P., Li J., Li Q. X. (2012). Simultaneous heterotrophic nitrification and aerobic denitrification by bacterium *Rhodococcus* sp. CPZ24. *Bioresource Technology*.

[24] Zheng H.-Y., Liu Y., Gao X.-Y., Ai G.-M., Miao L.-L., Liu Z.-P. (2012). Characterization of a marine origin aerobic nitrifying-denitrifying bacterium. *Journal of Bioscience and Bioengineering*.

[25] Qu D., Wang C., Wang Y., Zhou R., Ren H. (2015). Heterotrophic nitrification and aerobic denitrification by a novel groundwater origin cold-adapted bacterium at low temperatures. *RSC Advances*.

[26] Ji B., Yang K., Wang H., Zhou J., Zhang H. (2015). Aerobic denitrification by *Pseudomonas stutzeri* C3 incapable of heterotrophic nitrification. *Bioprocess and Biosystems Engineering*.

[27] He T., Li Z., Sun Q., Xu Y., Ye Q. (2016). Heterotrophic nitrification and aerobic denitrification by *Pseudomonas tolaasii* Y-11 without nitrite accumulation during nitrogen conversion. *Bioresource Technology*.

[28] Su W., Zhang L., Li D., Zhan G., Qian J., Tao Y. (2012). Dissimilatory nitrate reduction by *Pseudomonas alcaliphila* with an electrode as the sole electron donor. *Biotechnology and Bioengineering*.

[29] Wan C., Yang X., Lee D.-J., Du M., Wan F., Chen C. (2011). Aerobic denitrification by novel isolated strain using NO_2_^−^-N as nitrogen source. *Bioresource Technology*.

[30] Frette L., Gejlsbjerg B., Westermann P. (1997). Aerobic denitrifiers isolated from an alternating activated sludge system. *FEMS Microbiology Ecology*.

[31] Zhao B., He Y. L., Zhang X. F. (2010). Nitrogen removal capability through simultaneous heterotrophic nitrification and aerobic denitrification by *Bacillus* sp. LY. *Environmental Technology*.

[32] Sun Y., Li A., Zhang X., Ma F. (2015). Regulation of dissolved oxygen from accumulated nitrite during the heterotrophic nitrification and aerobic denitrification of *Pseudomonas stutzeri* T13. *Applied Microbiology and Biotechnology*.

[33] Jin R., Liu T., Liu G., Zhou J., Huang J., Wang A. (2014). Simultaneous heterotrophic nitrification and aerobic denitrification by the marine origin bacterium *Pseudomonas* sp. ADN-42. *Applied Biochemistry and Biotechnology*.

[34] Jiang P., Zhao C.-G., Jia Y.-Q., Yang S.-P. (2015). Effects of nitrite on ammonia-nitrogen removal and nitrite-nitrogen as well as photopigment biosynthesis of *Marichromatium gracile* YL28. *Microbiology China*.

[35] Padhi S. K., Tripathy S., Sen R., Mahapatra A. S., Mohanty S., Maiti N. K. (2013). Characterisation of heterotrophic nitrifying and aerobic denitrifying Klebsiella pneumoniae CF-S9 strain for bioremediation of wastewater. *International Biodeterioration and Biodegradation*.

[36] Zhang P.-Y., Qu Y., Yu D.-S., Guo S.-S., Yang R.-X. (2010). Comparison of heterotrophic nitrification and aerobic denitrification system by strain qy37 and its accelerating removal characteristic of NH_4_^+^-N. *Huanjing Kexue/Environmental Science*.

[37] van Niel E. W. J., Braber K. J., Robertson L. A., Kuenen J. G. (1992). Heterotrophic nitrification and aerobic denitrification in *Alcaligenes faecalis* strain TUD. *Antonie van Leeuwenhoek*.

[38] Zhang Y., Shi Z., Chen M., Dong X., Zhou J. (2015). Evaluation of simultaneous nitrification and denitrification under controlled conditions by an aerobic denitrifier culture. *Bioresource Technology*.

